# Mechanism of LCN2 in cerebral ischemia-reperfusion injury

**DOI:** 10.3389/fnins.2025.1536055

**Published:** 2025-03-20

**Authors:** Luo-yang Cai, Ying Yuan, Hai Huang, Jin Zhang, Xin-yi Zou, Xiao-ming Zhang

**Affiliations:** ^1^School of Acupuncture-Moxibustion and Orthopedics, Hubei University of Chinese Medicine, Wuhan, China; ^2^Hubei Shizhen Laboratory, Wuhan, China; ^3^Hubei University of Chinese Medicine, Wuhan, China; ^4^Hubei Provincial Hospital of Traditional Chinese Medicine, Wuhan, China; ^5^Affiliated Hospital of Hubei University of Traditional Chinese Medicine, Wuhan, China; ^6^Sub-Health Institute Hubei University of Chinese Medicine, Wuhan, China

**Keywords:** cerebral ischemia-reperfusion injury, lipocalin-2, inflammatory response, astrocyte, apoptosis

## Abstract

Cerebral ischemia-reperfusion injury (CIRI) is a complex pathophysiological process faced by brain tissues after ischemic stroke treatment, which involves mechanisms of inflammatory response, oxidative stress and apoptosis, and severely affects treatment outcome. Lipocalin-2 (LCN2), an acute-phase protein, is significantly up-regulated after CIRI and promotes neural repair by enhancing astrocyte phagocytosis, but its over-activation may also trigger secondary inflammation and demyelination injury. LCN2 also plays a key role in neuroinflammation regulation by regulating the polarization state of astrocytes and the release of inflammatory factors, and may affect the integrity of the blood–brain barrier and a variety of pathologic injury processes. In view of the important role of LCN2 in CIRI, this article reviews the mechanism of LCN2, aiming to provide new ideas and methods for the treatment of ischemic stroke.

## 1 Introduction

Cerebral ischemia-reperfusion injury (CIRI) is a phenomenon in which brain tissue undergoes further damage due to a series of complex biochemical and molecular biological changes when blood supply is restored after ischemia, a process that involves complex pathophysiological mechanisms including inflammatory response, oxidative stress, and apoptosis (Tuo et al., 2022). This injury not only exacerbates neuronal cell death, but also may trigger more extensive brain tissue dysfunction. Currently, despite the success of thrombolysis and thrombectomy in the treatment of acute ischemic stroke, reperfusion injury is still an important factor affecting the treatment outcome (Zhang et al., 2022). Therefore, in-depth study of the molecular mechanisms of cerebral ischemia-reperfusion injury and searching for effective therapeutic targets are of great significance for improving the therapeutic outcome of ischemic stroke.

Lipocalin-2 (LCN2), a member of the lipid carrier protein family, functions as an acute-phase protein after brain injury, and LCN2, an important marker of reactive astrocytes, is significantly up-regulated after cerebral ischemia-reperfusion injury, which promotes neurological repair by enhancing phagocytosis of astrocytes and removing damaged tissue debris (Jung and Ryu, 2023). However, excessive phagocytic activation may also lead to secondary inflammatory responses and demyelination injury, suggesting a delicate balance between LCN2’s role in regulating inflammatory responses and cytoprotection (Zhao et al., 2023). Second, LCN2 plays a key role in the regulation of neuroinflammation by affecting the polarization state of astrocytes and regulating the type of inflammatory factors they release, a process that not only affects the extent of local brain tissue damage, but may also further modulate the systemic inflammatory response by affecting the integrity of the blood–brain barrier, as well as participating in a variety of pathological damage processes such as oxidative stress and neuronal apoptosis (Zhang et al., 2022; Tan et al., 2024). Given that the pathophysiological mechanism of cerebral ischemia-reperfusion injury involves a number of aspects mentioned above, we hypothesize that LCN2 may play an important role in this process. This article comprehensively reviews the mechanism of LCN2 in cerebral ischemia/reperfusion injury, not only aims to provide new ideas and methods for the treatment of ischemic stroke, but also hopes to explore the possibilities of LCN2 as a potential therapeutic target by systematically sorting out the results of the existing research, so as to lay a solid theoretical foundation for the subsequent preclinical studies and clinical trials. Further, by clarifying the specific pathway of LCN2 in cerebral ischemia/reperfusion injury, we can help to develop more targeted therapeutic drugs with fewer side effects and optimize the clinical therapeutic regimen to improve the quality of patients’ survival and prognosis. Meanwhile, a deeper understanding of the mechanism of LCN2 can also enrich the theoretical system of cerebral ischemia/reperfusion injury and provide new perspectives and directions for basic research in this field.

## 2 Mechanisms of cerebral ischemia-reperfusion injury

### 2.1 Oxidative stress

Oxidative stress is one of the core mechanisms of cerebral ischemia-reperfusion injury. Under ischemia, brain cells are unable to carry out normal aerobic metabolism, leading to disorders in energy metabolism (Qin et al., 2022). When the blood supply is restored, a large influx of oxygen triggers an abnormal oxidative phosphorylation process, generating a large number of reactive oxygen species (ROS) and free radicals, such as superoxide anion and hydroxyl radicals, etc. (Chen et al., 2020). These highly reactive molecules undergo oxidative reactions with biomolecules, such as cell membranes, proteins, and nucleic acids, leading to severe damage to the structure and function of the cells. Serious damage to cell structure and function (Granger and Kvietys, 2015; Sies, 2015). Oxidative stress not only directly destroys cellular components, but also activates a series of downstream signaling pathways that further exacerbate cell damage and death (Zhu et al., 2022).

### 2.2 Inflammatory response

Inflammatory response is a key factor leading to neurological impairment after cerebral ischemia and reperfusion. During ischemia, damaged cells release inflammatory factors such as tumor necrosis factor-α (TNF-α), which attracts immune cells to gather in the ischemic area (Li et al., 2023). Upon reperfusion, microglia and astrocytes further activate and release large amounts of inflammatory mediators, leading to an increased local inflammatory response (Mao et al., 2023). The inflammatory response not only directly damages brain tissue, but also creates a vicious cycle by destroying the blood–brain barrier, allowing harmful substances and inflammatory cells in the blood to enter brain tissue (Levard et al., 2021). In addition, the inflammatory response activates the complement system, exacerbating microvascular injury and thrombosis, further worsening ischemia-reperfusion injury.

### 2.3 Calcium ion overload

Calcium ion overload is another important mechanism of cerebral ischemia-reperfusion injury. Under ischemia, cell membrane permeability increases and calcium ion channels open, leading to a large inward flow of extracellular calcium ions. When reperfusion occurs, due to the impairment of the intracellular calcium ion clearance mechanism, calcium ions further accumulate, forming calcium overload (Zhang et al., 2019). Calcium overload not only directly destroys the cytoskeleton and membrane structure, but also activates a series of calcium-dependent enzymes, such as phospholipases and proteases, which promote the degradation of membrane phospholipids and the breakdown of cellular structural proteins (Cataldi, 2013). Under calcium overload, calmodulin (CaM) binds to calcium ions in complexes increasing the release of vasoconstrictor factors, causing vasospastic constriction affecting blood flow and exacerbating ischemic–hypoxic injury after CIRI (Ludhiadch et al., 2022). Accumulation of intracellular calcium ions in mitochondria leads to damage to the mitochondrial membrane, inhibiting ATP synthesis, which in turn leads to impaired energy synthesis, and these changes ultimately lead to apoptosis and necrosis, which exacerbate the damage to brain tissue (Rahi and Kaundal, 2024; Bodalia et al., 2013).

### 2.4 Apoptosis

Apoptosis is an important form of cell death in cerebral ischemia-reperfusion injury. During ischemia/reperfusion, the above factors work together to activate apoptotic signaling pathways, which induce orderly cell death by regulating the expression of apoptosis-related genes and the activation of apoptosis proteins (Momenabadi et al., 2022; Wang et al., 2020). The B-cell lymphoma (Bcl) family plays an important role in apoptosis, which includes the anti-apoptotic gene Bcl-2 and the pro-apoptotic gene Bcl-2-associated X protein (Bax) (Yang et al., 2022). Apoptosis involving the Bcl family usually works through cystatinases (Caspases) mediating protein cleavage, and when CIRI occurs, Caspase-3 is activated, inducing apoptosis in neuronal cells, resulting in further damage to neuronal cells (Niizuma et al., 2010). In addition, the cellular contents released by apoptotic cells may further activate the inflammatory response, forming a vicious cycle and exacerbating brain tissue damage and dysfunction (Chen et al., 2011).

In summary, oxidative stress, inflammatory response, calcium ion overload and apoptosis are the main mechanisms of cerebral ischemia-reperfusion injury. These mechanisms are interrelated and interact with each other, and together they lead to severe injury and dysfunction of brain tissue. Therefore, therapeutic strategies targeting these mechanisms are important for reducing cerebral ischemia-reperfusion injury and promoting neurological function recovery.

## 3 Structure and function of LCN2

### 3.1 Structure

LCN2 is a small-molecule protein whose structure consists mainly of β-folding to form a barrel-like structure known as the β-folding barrel, and one end of this β-folding barrel is closed by a short N-terminal 310-helix, while the other end is open for ligand binding (Jaberi et al., 2021). The β-folding barrel of LCN2 consists of multiple antiparallel β-folds that are interconnected by interchain hydrogen bonds to form a stable structure (Chandrasekaran et al., 2024). Hydrophobic aromatic and aliphatic amino acid residues are arranged in the interior of the barrel structure to form a hydrophobic nucleus, which provides a site for the binding of lipophilic ligands that have specific chemical and physical properties to bind to specific ligands (e.g., iron ions, small molecule metabolites, etc.) (Schröder et al., 2023). Upon binding a ligand, the structure of LCN2 may undergo minor changes to adapt to the shape and charge of the ligand (Li et al., 2020). The structure of LCN2 may also contain specific functional regions that may be involved in interactions with other proteins or cellular receptors to regulate cell signaling or cell–cell interactions (Krizanac et al., 2024).

### 3.2 Function

**Figure 1:**
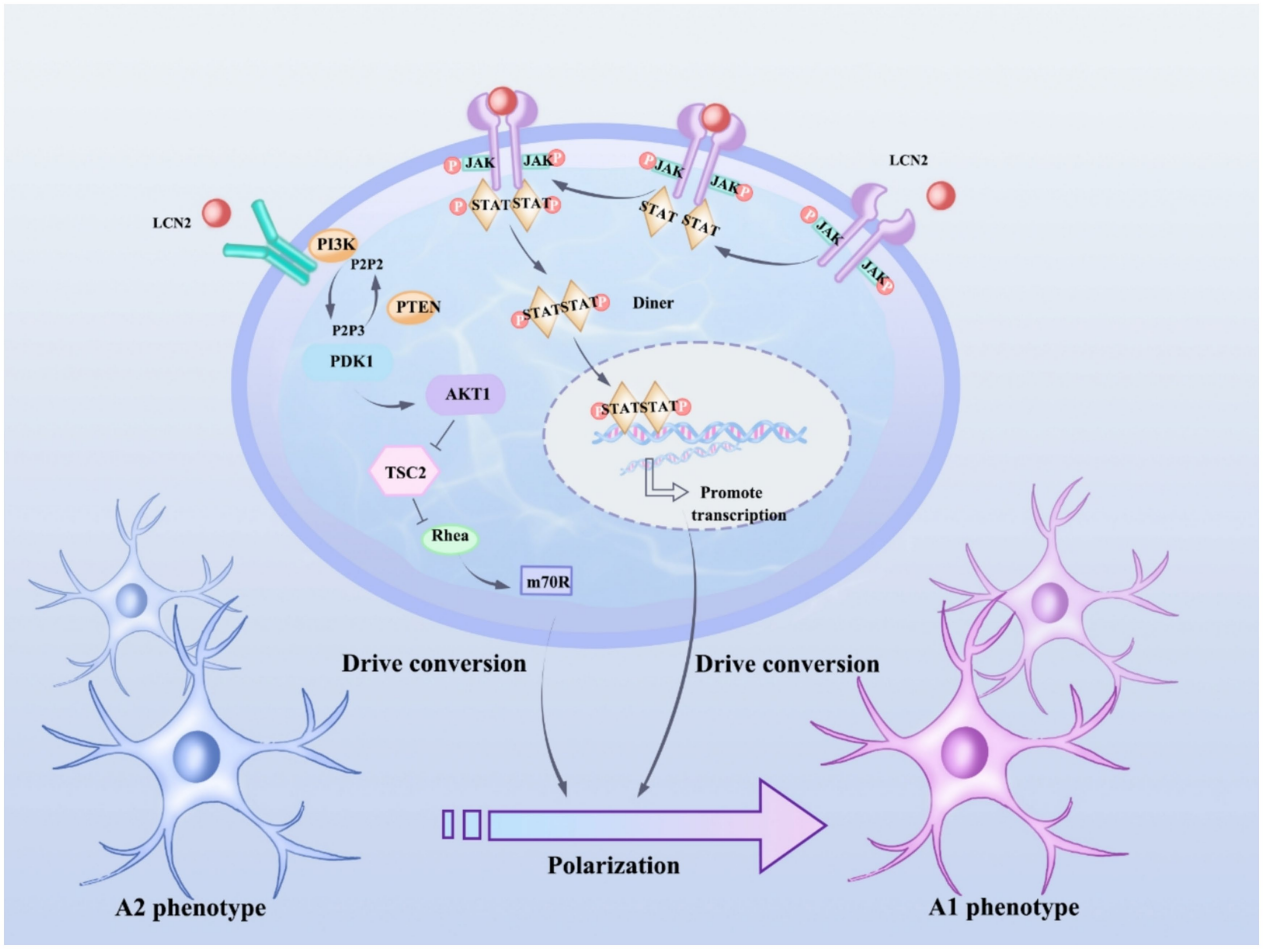
LCN2 affects astrocyte polarization status by regulating JAK/STAT and PI3K/Akt pathways. JAK activation phosphorylates STAT proteins, and phosphorylated STAT3 forms a dimer and translocates to the nucleus, where the STAT3 dimer binds to the promoter regions of genes associated with astrocyte polarization and promotes gene transcription, thus promoting the shift from an anti-inflammatory phenotype (type A2) to a pro-inflammatory phenotype (type A1) in astrocytes. After activation of PI3K, phosphatidylinositol-3,4,5-trisphosphate (PIP3) is produced, which in turn activates Akt, which regulates a variety of downstream targets, including tuberous sclerosis complex 2 (TSC2) and mammalian target of rapamycin (mTOR), which play important roles in cell metabolism and the synthesis of polarization-associated proteins, and thus drives the transformation of astrocytes from A2 to A1 phenotypes.

LCN2 is expressed in a variety of cell types, such as neutrophils, endothelial cells, smooth muscle cells, cardiomyocytes, and macrophages (Tarín et al., 2016)^.^ LCN2 is widely present in human tissues, but its expression is low, and is only elevated when epithelial cells are stimulated by infection, inflammation, and ischemia, and is involved in inflammation, lipid metabolism, iron transport, and renal tubular repair (Rehwald et al., 2020; Zhang et al., 2019). Recent studies show that LCN2 is an autocrine promoter of chemokine inducers and reactive astrocytosis (Jang et al., 2013). Astrocytes can undergo functional polarization under pathological conditions and exhibit either classically activated or alternatively activated phenotypes, and LCN2 may regulate the polarization state of astrocytes by affecting their signal transduction pathways (Ni et al., 2015) (Figure 1). Specifically, LCN2 may promote the conversion of astrocytes to a classically activated phenotype, release pro-inflammatory factors, and exacerbate the inflammatory response; however, under certain conditions, LCN2 may also induce the conversion of astrocytes to an alternatively activated phenotype, release anti-inflammatory factors, and promote tissue repair (Wang et al., 2021; Liu et al., 2024; Zhou et al., 2024).

**Figure 2:**
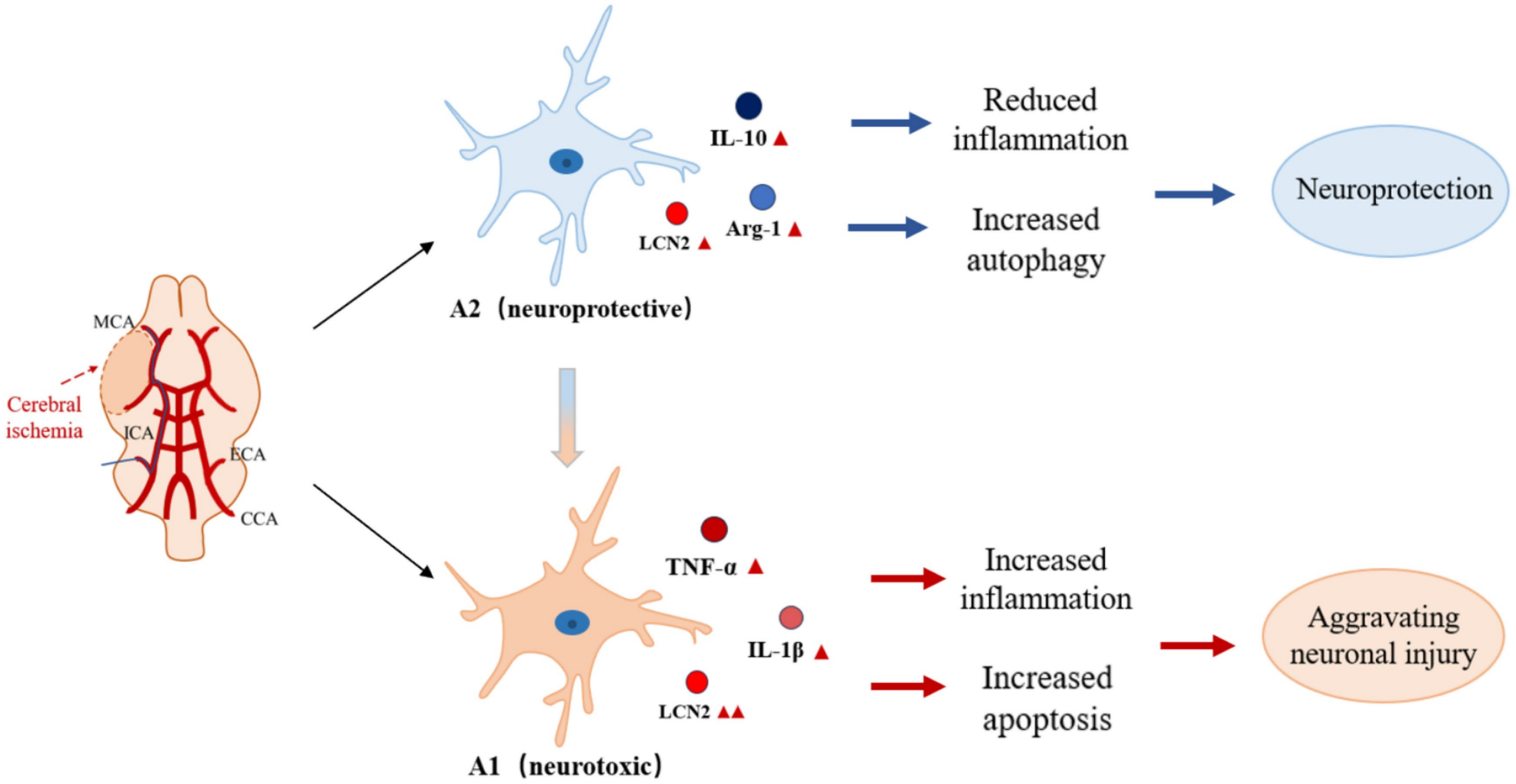
Role of LCN2 in CIRI by regulating astrocyte activation state. Under pathological conditions such as cerebral ischemia-reperfusion injury, astrocytes are activated and express LCN2. LCN2 enhances phagocytosis in astrocytes by activating downstream phagocytosis signaling pathways through binding to receptors mediating phagocytosis, a process that may play an important role in removing debris from damaged tissues and promoting tissue repair. However, over-activation of LCN2 may lead to inflammatory responses and secondary injury. As an important inflammatory mediator in the central nervous system, LCN2 can promote the shift of astrocytes from anti-inflammatory to pro-inflammatory phenotype, participate in the regulation of neuroinflammation, and exacerbate neuronal damage and death by promoting oxidative stress, mitochondrial dysfunction, and other mechanisms.

Under pathological conditions such as cerebral ischemia-reperfusion injury, astrocytes are activated and express LCN2. LCN2 activates downstream phagocytic signaling pathways by binding to receptors that mediate phagocytosis, such as LRP1, which enhances phagocytosis by astrocytes, a process that may play an important role in removing debris from damaged tissues and facilitating tissue repair, but may also lead to excessive inflammatory responses and secondary damage (Jha et al., 2015) (Figure 2). LCN2, an important inflammatory mediator in the central nervous system, induces the activation of astrocytes and microglia, releases pro-inflammatory factors, and participates in the regulation of neuroinflammation (Jung et al., 2023). In a variety of neurodegenerative lesions, aberrant expression of LCN2 may be associated with degeneration of dopaminergic neurons and decline in cognitive function, and LCN2 may exacerbate neuronal damage and death through mechanisms that promote oxidative stress and mitochondrial dysfunction (Bi et al., 2013). In a variety of CNS injuries (e.g., cerebral ischemia, cerebral hemorrhage, traumatic brain injury, etc.), reactive astrocytes characteristically express LCN2, which can therefore be used as a marker of reactive astrocytes for assessing the extent and prognosis of CNS injury (Liu et al., 2022). In addition, the expression level of LCN2 may serve as a diagnostic indicator for certain neurological disorders and as a monitor of treatment efficacy (Suk, 2016).

In summary, LCN2 associated with astrocytes has multiple functions and plays an important role in the physiological and pathological processes of the central nervous system. Further studies on the functions and mechanisms of LCN2 can help to reveal the pathogenesis of neurological diseases and provide new targets and strategies for their treatment.

## 4 Mechanisms of LCN2 in cerebral ischemia-reperfusion injury

### 4.1 Astrocyte activation

One study revealed the important role of LCN2 in regulating the activation state of astrocytes, and they found that LCN2 treatment significantly promoted astrocytes’ expression of cytokines closely related to the classical activation pathway, such as IL-1β, TNF-α, inducible nitric oxide synthase (iNOS), and CXCL10 (Wang et al., 2015; Adler et al., 2023). In contrast, LCN2 treatment did not induce the expression of alternative activation-associated cytokines such as Arg1, IL-10, MRC1, YM1, and Fizz1 (Zhao et al., 2019). This finding further emphasizes the specific role of LCN2 in regulating the activation state of astrocytes. In addition, studies have identified a critical role for LCN2 in lipopolysaccharide (LPS)/interferon gamma (IFN-γ)-induced classical activation of astrocytes, and provide strong evidence to support this idea (Kim et al., 2017). Their study shows that LCN2 is not only an important regulator of astrocyte classical activation after ischemic stroke, but also likely a key determinant of its activation status.

The neurotoxic and neuroprotective effects exhibited by LCN2 in cerebral ischemia/reperfusion injury depend largely on the timing and conditions of injury. It has been shown that iNOS expression is significantly impaired in astrocytes knocked down for LCN2, which highlights the critical role of LCN2 in promoting the classical activation of the astrocyte pathway, suggesting that it may be one of the key factors exacerbating cerebral ischemia/reperfusion injury under certain conditions (Zhao et al., 2019). Ranjbar Taklimie et al. (2019) cultured astrocytes from the cerebral cortex of mice under hypoxic conditions and observed that the level of LCN2 gradually increased with the duration of hypoxia, especially reaching a peak at 24 and 72 h, and that LCN2 was able to promote a shift from an anti-inflammatory phenotype to a pro-inflammatory phenotype of astrocytes, which further exacerbated cerebral ischemia-reperfusion injury. In addition, Wang et al. (2015) conducted an in-depth study using a rat middle cerebral artery infarction model and found that after the onset of cerebral ischemia/reperfusion injury, the expression of LCN2 was gradually elevated mainly in astrocytes and endothelial cells, and that this elevation reached a peak 24 h after cerebral infarction in rats. This suggests that changes in LCN2 expression are closely related to the development of injury at specific time nodes after the onset of cerebral ischemia-reperfusion injury, and that LCN2 exhibits more neurotoxic effects at this critical time.

In terms of injury conditions, numerous findings have shown that LCN2 can exert neurotoxic effects after the onset of IS. The underlying mechanism may be that LCN2 up-regulates glial fibrillary acidic protein (GFAP) produced during astrocyte activation through the Ras homolog-Rho-associated helical coiled-coil protein kinase signaling pathway, which leads to morphological changes of astrocytes in response to inflammatory stimuli, and then causes neurological damage (Mao et al., 2016; Jin et al., 2014). This suggests that under the specific conditions of ischemic injury, LCN2 triggers neurotoxicity through specific signaling pathways. However, it has also been shown that in the early stage of brain injury, LCN2 can promote neural repair by enhancing phagocytosis of astrocytes and removing damaged tissue fragments, reflecting a neuroprotective effect. This suggests that the function of LCN2 changes under different injury timing and conditions, and that its neurotoxic and neuroprotective effects are not absolute, but are dynamically regulated by a variety of factors. The in-depth study of these relationships is of great significance to the understanding of the pathological mechanisms of cerebral ischemia/reperfusion injury and the search for precise therapeutic strategies for LCN2.

### 4.2 Oxidative stress

After cerebral ischemia-reperfusion, LCN2 expression is up-regulated. On the one hand, LCN2 may inhibit the gene transcription of antioxidant enzymes, such as SOD, GPx, etc., by binding to specific transcription factors in the promoter region of antioxidant enzyme genes, or accelerate protein degradation of antioxidant enzymes through the ubiquitin-proteasome pathway, which reduces the cell’s scavenging ability of free radicals; on the other hand, in the metabolism of ferric ions, LCN2 can combine with iron ions to form a complex, which affects the intracellular transport and storage of iron ions (Wang et al., 2022; Yamada et al., 2016). For example, LCN2 may promote the expression of transferrin receptor 1 (TfR1), which increases cellular uptake of iron ions, resulting in elevated intracellular free iron ion concentrations (Wang et al., 2024). In the Fenton reaction, iron ions act as catalysts to promote the decomposition of hydrogen peroxide to produce highly reactive hydroxyl radicals; the Haber-Weiss reaction also accelerates the generation of free radicals due to the involvement of iron ions, further exacerbating the oxidative stress (Fei et al., 2024). In addition, LCN2, as a lipid transporter protein, can bind to phospholipid molecules in the cell membrane, altering the lipid bilayer structure of the cell membrane, making the unsaturated fatty acids of the membrane phospholipids more susceptible to attack by free radicals, forming lipid peroxidation products, destabilizing cell membranes, and increasing the sensitivity of cells to oxidative stress (Ferreira et al., 2018; Ferreira et al., 2018). In summary, LCN2 plays an important regulatory role in the oxidative stress process in cerebral ischemia-reperfusion injury, and it exacerbates the generation of free radicals and cellular damage by regulating the activity of antioxidant enzymes, influencing iron metabolism, and altering the lipid composition of cell membranes.

## 5 Conclusion

Compared with other common biomarkers in ischemic stroke, such as neuron-specific enolase (NSE) and S100 calcium-binding protein B (S100B), LCN2 has unique diagnostic and therapeutic potential. NSE is mainly found in neurons and neuroendocrine cells (Persson et al., 1987). After ischemic stroke, neuronal damage leads to the release of NSE into the blood, and its serum level may rise within hours after the onset of the disease, which is valuable for early diagnosis, but its specificity is relatively limited (Isgrò et al., 2015). S100B is mainly secreted by astrocytes and enters the blood circulation when the blood–brain barrier is damaged after brain injury. Changes in its level can reflect the degree of brain injury, but it is also affected by a variety of factors, such as peripheral tissue damage (Lamers et al., 2003). LCN2, as an important marker of reactive astrocytes, was significantly upregulated after cerebral ischemia-reperfusion injury, which not only reflects the activation status of astrocytes, but also participates in a number of pathological processes such as inflammatory response, oxidative stress, and neuronal apoptosis (Wan et al., 2022). This study reviews the recent research progress of LCN2 in cerebral ischemia-reperfusion injury, revealing that LCN2 is involved in the functional regulation of astrocytes through multiple mechanisms, which in turn affects the injury and repair process of brain tissue. This finding provides potential molecular targets for the development of novel therapeutic strategies against cerebral ischemia/reperfusion injury.

Although a large number of studies have supported the pathological role of LCN2 in cerebral ischemia/reperfusion injury, some studies have suggested a different viewpoint. Some studies suggest that LCN2 may have neuroprotective effects under certain conditions. In the early stage of cerebral ischemia/reperfusion injury, low levels of LCN2 may help to maintain neuronal survival and function by activating specific intracellular signaling pathways, promoting the conversion of astrocytes to A2 type, and releasing neuroprotective factors such as brain-derived neurotrophic factor (BDNF) (Xiao et al., 2025). It has also been found that LCN2 may be involved in the body’s self-repair mechanism, and that the up-regulation of LCN2 expression during a certain period of time after injury can promote the removal of necrotic tissues and apoptotic cells by phagocytes and accelerate the repair process of the injured area (Liu et al., 2018).

From the perspective of therapeutic significance and potential clinical application, it is crucial to deeply investigate the mechanism of LCN2. Clarifying the interactions between LCN2 and other inflammatory factors can help to understand the inflammatory network and develop therapeutic tools to comprehensively regulate inflammation; and realizing the precise regulation of LCN2 levels can intervene in the damage process from the source. In addition, the development of new drugs targeting LCN2 is promising, which can inhibit its overexpression in the neurotoxic stage and promote its function in the neuroprotective stage. Meanwhile, the development of new diagnostic markers based on LCN2 can provide a more accurate assessment of disease conditions and prognosis, and provide strong support for clinical treatment decisions.

Although this study reviewed the multiple mechanisms of LCN2 in cerebral ischemia-reperfusion injury, there are still some limitations, and further in-depth studies are needed to investigate the interaction mechanism between LCN2 and other inflammatory factors, its specific mode of action in different pathological stages, and how to achieve the best therapeutic effect by precisely regulating the level of LCN2, etc. In the future, more studies are needed to reveal the role of LCN2 in cerebral ischemia-reperfusion injury comprehensively and objectively, providing a more accurate theoretical basis for clinical treatment.
